# Purification of Dye-stuff Contained Wastewater by a Hybrid Adsorption-Periphyton Reactor (HAPR): Performance and Mechanisms

**DOI:** 10.1038/s41598-017-10255-8

**Published:** 2017-08-29

**Authors:** Yanfang Feng, Lihong Xue, Jingjing Duan, Dionysios D. Dionysiou, Yudong Chen, Linzhang Yang, Zhi Guo

**Affiliations:** 1 Key Laboratory of Agro-Environment in Downstream of Yangtze Plain, Ministry of Agriculture, Institute of Agricultural Resources and Environment, Jiangsu Academy of Agricultural Sciences, Nanjing, 210014 China; 2 State Key Laboratory of Soil and Sustainable Agriculture, Institute of Soil Science, Chinese Academy of Sciences, Nanjing, 210008 China; 3 Nanjing Institute of Environmental Science, MEP, Nanjing, 210042 China; 4 Stockbridge School of Agriculture, University of Massachusetts, Amherst, MA 01003 USA; 5 Environmental Engineering and Science Program, University of Cincinnati, Cincinnati, OH 45221-0071 USA

## Abstract

The aim of this study was to develop an environmental-benign bio-measure that could be used to purify dye-contaminated wastewater. Herein, a hybrid adsorption-periphyton reactor (HAPR), combining a bioadsorbent based adsorption unit and a periphyton-based photo-bioreactor (PPBR), was built and applied for the first time. Firstly, an efficient bioadsorbent, i.e, microwave-activated swede rape hull (MSRH), was produced, characterized and applied in an adsorption column, to pretreat methylene blue (MB) wastewater with high concentration (~100 mg L^−1^ or higher). Thereafter, the effluent of adsorption column, with lower dye concentration (~0.5 mg L^−1^), was collected and further purified by PPBR. Results showed that dye removal efficiency by HAPR was 99.95% (from 200 mg L^−1^ to 0.1 mg L^−1^ or lower). Decolorization mechanisms by PPBR, included adsorption process by extracellular polymeric substances (EPS) on the surface of periphyton and degradation process. The study showed that HAPR was a novel, environmental friendly, efficient and promising dye-purification method and deserved further attention in future investigation.

## Introduction

Annual worldwide production of synthetic dyes reaches 7 × 10^7^ tons, and around 10^5^ tons of synthetic dyes are released to aquatic systems^1^. Such colored effluents are highly toxic and carcinogenic to flora and fauna as well as humans^2, 3^. Among the already developed technologies, adsorption technology based on various adsorbents, such as activated carbons (ACs), are widely employed for the removal of dye-stuff from aqueous solutions^4^.

However, the application of ACs in large-scale faced some problems such as high cost, difficulty in regeneration^5^ and easy clogging. To lower the cost of adsorbents (such as ACs), many low-cost bioadsorbents have been developed and investigated^6, 7^. Microwave-assisted activation method avoids environmental side effects and reduces processing time, thus saving energy consumption and eliminating gas production^8^. Consequently, in this study, swede rape hull was selected and activated using microwave-assisted thermal treatment. Then an efficient low-cost bioadsorbent (microwave-activated swede rape hull, MSRH) was produced and applied in the first treatment unit.

Previous investigations showed that the application of adsorbents alone (e.g., activated carbons, or low-cost bioadsorbents), when applied at economical dosages, could not completely remove the color or reduce the contamination level to an acceptable standard^2, 6, 9^. Such not sufficiently treated wastewater is unacceptable for direct discharge to water bodies, because the presence of trace concentration of dyes in effluent is highly visible, undesirable, and may still have residual toxicity. This low concentration of dye-contained effluents is a new challenge in the field of remediation of colored wastewater effluents. Consequently, such wastewaters need to be further purified by certain cost-effective and environmental-friendly sustainable processes.

A few previous studies selected some microbial species to purify low-level dye-contained wastewater. For example, Waghmode^10^ reported the biodegradation of disperse dye Brown 3 REL using *Galactomyces geotrichum* and *Brevibacillus laterosporus*. But the application of single species usually has limitations, especially when the concentration of pollutants is high. Shabbir reported the degradation of food dye by epiphyton, metaphyton and epilithon^1^, but this kind of chemical compound is less toxic to aquatic organisms, and further studies are needed for some toxic dye compounds.

Consequently, to solve the above problems, environmental-friendly measures based on microbial aggregates have aroused interests in removing pollutants from aquatic systems^11–13^. Periphyton, which is composed of autotrophic and heterotrophic microorganisms, is a kind of microbial aggregates attached on submerged surface in almost every natural water bodies^14–17^. These complex aggregates are usually highly tolerant to toxic environment and highly effective in removing various contaminants such as microcystin-RR, Cr and Cd^18–20^. Moreover, the organisms of periphyton are usually coated with an “organic shell” such as extracellular polymeric substances (EPS), which may have an efficient pollutant-binding capacity and biological activity for dye molecules^11^.

However, the colored effluents of printing and dyeing industry are usually heavily polluted^21^, which could not be directly purified by living organisms such as periphyton, consequently, a pretreatment is necessary. Considering the significance and practicality of including a self-cleaning and renewable bio-based system to remove contaminants such as dye-stuff, a hybrid adsorption-periphyton reactor (HAPR), combining an adsorption unit and a biological treatment unit, was designed and investigated. Specifically, HAPR includes an adsorption column based on a low-cost efficient bioadsorbents (MSRH) to pretreat the high concentrations of dye wastewater and a periphyton based photo-bioreactor (PPBR) to degrade the residual dyes present at low concentration as discharged from the first adsorption unit.

In summary, the objectives of this study were to: (1) develop a hybrid adsorption-periphyton reactor (HAPR) to enhance the removal efficiency of a model cationic dye (Methylene Blue, MB) from wastewater, and (2) investigate the removal mechanism of dye-stuff by HAPR. The study will provide an environmentally benign measure to enhance the purification of dye wastewater and give an insight into MB removal mechanisms by periphyton and provides possible suggestions to enhance dye purification by modulating the adsorption process.

## Materials and Methods

### The preparation of bioadsorbent (MSRH)

Crude swede rape hull (*B*. *napus* L.) was collected in Shandong province, China. Natural sunlight dried hull was washed by deionized water and dried in an oven at 60 °C. Then the dried biomass was crushed, sieved (pore diameter was 250 μm), and activated by a microwave oven (Galanz wp800sl23-2, China). Briefly, the activation procedure was as follows: (1) crude swede rape hull powder was scattered in a glass plate with the thickness of around 2–5 mm and the glass plate together with the biomass powder was placed in the microwave oven; (2) the activation was conducted at high power (800 W) for 2 min; then the samples were cooled for 1 min; (3) the activation-cooling procedure was repeated 3 times. The microwave-activated swede rape hull (MSRH) was then produced and stored in an air tight glass bottle for further use.

### The description of the Hybrid Adsorption-Periphyton Reactor (HAPR)

**Figure 1:**
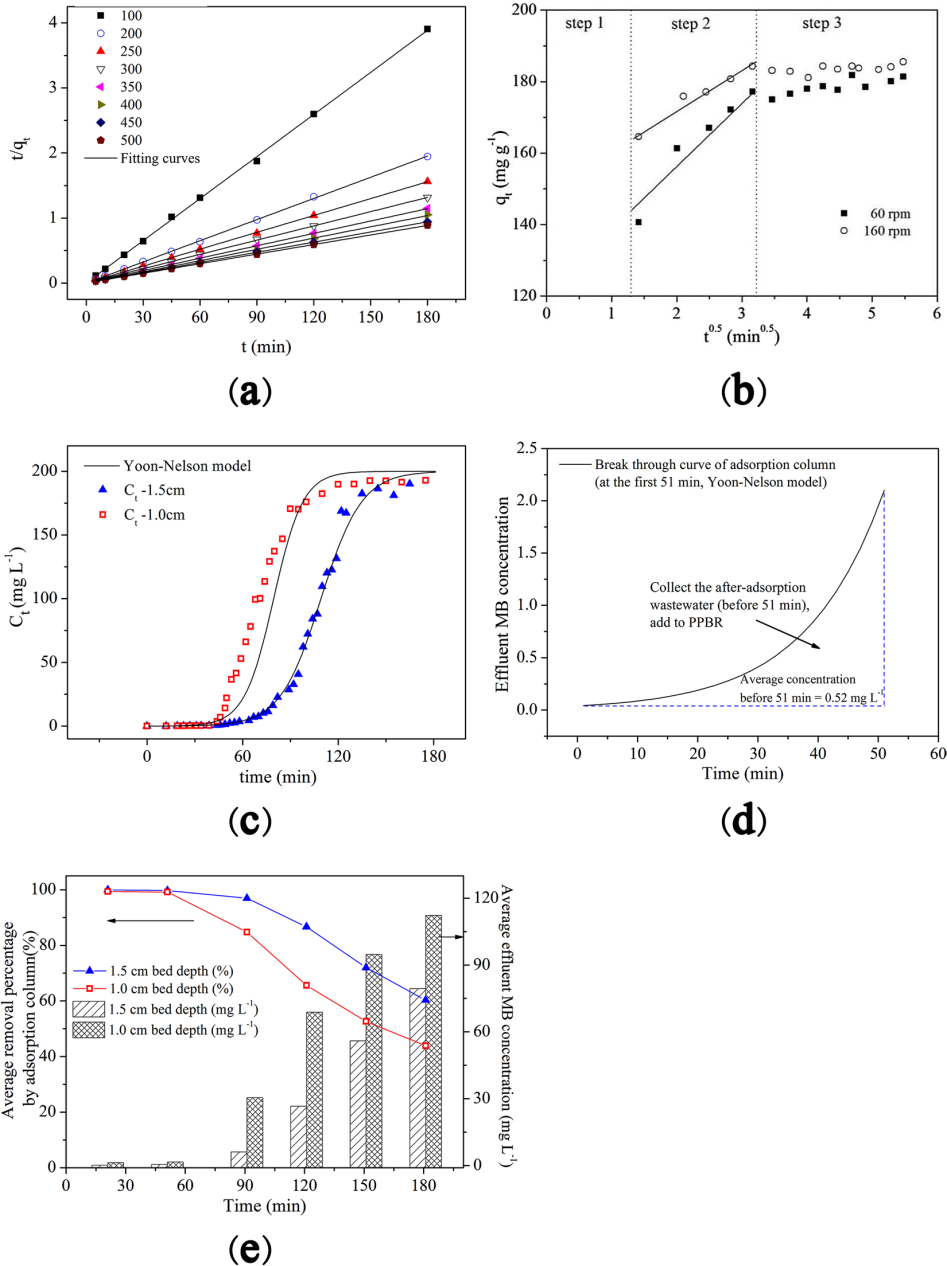
(**a**) Plots for pseudo-second kinetic model in batch experiment; **(b)** Plots for intra-particle diffusion model in batch experiment; **(c)** Breakthrough curves of adsorption column for MB adsorption onto MSRH at different bed depth (flow rate = 10.16 mL min^−1^, initial dye concentration = 200 mg L^−1^, pH 5.50, bed depth = 1.0, 1.5 cm); (**d**) The prediction of average MB concentration of the effluents of adsorption column. (Conditions: bed depth = 1.5 cm, initial MB concentration = 200 mg L^−1^, flow rate = 10.16 mL min^−1^) and **(e)** The predicted average effluent MB concentration and removal percentage of MSRH-based adsorption column (initial inflow MB concentration = 200 mg L^−1^).

HAPR consisted of two units: MSRH-based adsorption column and periphyton-based photo-bioreactor (PPBR) (see Fig. 1S in Appendix 1). The adsorption column (Appendix 2) was filled with a certain amount of MSRH (bed depth = 1.5 cm), and to avoid the leakage of bioadsorbent particles, glass wool was placed at the column bottom. The PPBR was made of several glass containers with capacity of 5 L (Appendix 3). To collect and culture periphyton biofilms, 30% of the PPBR volume was filled with substrates (spec: 200 mm × 0.5 mm, Jineng Environmental Protection Company of Yixing, China). A piece of gauze was covered on the top of PPBR in order to prevent insects flying into the reactor. When periphyton grew to mid-logistic growth curve stage (i.e., 4 weeks after incubation), the Hybrid Adsorption-Periphyton Reactor was operated to conduct this study.

Influent with high concentration Methylene blue (C_16_H_18_ClN_3_S·3H_2_O, C.I.52015, MB) solution, with concentration of 200 mg L^−1^, was injected into the MSRH-based adsorption column and then the effluent was added to the PPBR. The hydraulic retention time (HRT) was 1.85 min and 48 h in the MSRH-based adsorption column and PPBR, respectively. In this study, to simulate low concentration MB discharge in practice and keep continuous running of the PPBR, MB solutions of low concentrations (0.2 and 0.5 mg L^−1^) were added to PPBR every 48 h. After treatment of MB wastewater by PPBR, water samples of MB-contained effluents were collected before next addition of MB wastewater. Specifically, MB solution with different concentrations (i.e., 0.2 and 0.5 mg L^−1^, respectively) were added to two independent PPBR systems at around 9:00 am in the first day, and the samples were collected at around 9:00 am in the third day. Thereafter, MB solutions were added and samples were collected every 48 hours. This process repeated at least 12 cycles.

### Characterization of bioadsorbent (MSRH)

The bioadsorbent was characterized by a scanning electron microscope (SEM) (Quanta 200, FEI, Netherlands) at magnification of 1000X, Fourier Transform Infrared Spectroscope (FTIR) (Nicolet 360, Thermo Electron Co., USA) at a spectral range of 4000 to 400 cm^−1^ and particle size distribution analyzer (Mastersizer 2000, Malvern Instruments, UK).

### Adsorption experiments for MSRH

The concentration of MB stock solution for adsorption experiment was 1000 mg L^−1^. The concentration of MB was measured at 664 nm using a UV-vis spectrophotometer (Shimadzu, UV2450, Japan); detailed information of analysis method was presented in our previous study^2^. The amounts of MB adsorbed onto the bioadsorbents at equilibrium and at time t were obtained by the following equations:

1 $${{\rm{q}}}_{{\rm{e}}}=\frac{({C}_{0}-{C}_{e})V}{m}$$

2 $${{\rm{q}}}_{{\rm{t}}}=\frac{({C}_{0}-{C}_{t})V}{m}.$$

### Model studies

Equilibrium models (such as Langmuir, Freundlich model) and kinetic models (such as pseudo-second-kinetic model and intraparticle diffusion model) were applied to describe the adsorption process. In addition, the performance of MSRH adsorption column operation was described by breakthrough curves to determine the operation and dynamic responses^22^. Relevant equations are presented in Appendix 4.

### Determination of microbial activity of periphyton in PPBR

In order to investigate the removal mechanisms of MB by periphyton in PPBR, determination of microbial activity by Biolog^TM^ ECO Microplates (Hayward, CA, USA) was performed. These Biolog^TM^ ECO Microplates, containing 96 wells and 31 types of carbon sources, were applied to characterize the microbial respiratory activity in periphyton biofilms. The wells contain a redox-sensitive tetrazolium dye (oxidation indicator) and turn purple as a result of respiratory electron transport in metabolically active cells^23^. Therefore, the different plate color is proportional to respiratory activity. Specifically, 150 μL prediluted periphyton-water mixture was added into each well of every Biolog^TM^ ECO Microplate, and was incubated at 25 °C. The absorbance was determined at 590 nm using a Biolog Microplate Reader at predetermined time intervals for several days. More detailed information is presented in Appendix 5.

### Determination of bioadsorption and biodegradation by periphyton in PPBR

NaN_3_ was used to suppress microbial respiration and inhibit the process of biodegradation. If the microbial activities of periphyton were completely inhibited by NaN_3_, the removal of MB under this condition was mainly due to adsorption process; on the contrary, if the periphyton was not inhibited, the removal of MB may be both due to adsorption and biodegradation. Consequently, if MB removal performance shows no differences between the inhibited and uninhibited periphyton, a conclusion should be made that the removal of MB by PPBR is mainly due to adsorption. Herein, the microbial activities of periphyton in PPBR were inhibited by adding 1% NaN_3_ (w/v). Then the removal percentage of MB by NaN_3_-inhibited periphyton and control (without NaN_3_) was studied at two initial dye concentrations (0.2 and 0.5 mg L^−1^).

## Results and Discussion

### The evaluation of dye removal process by MSRH

#### Equilibrium study

Langmuir model and Freundlich model are usually applied in isotherm adsorption studies. Langmuir model assumes that adsorption occurs on a homogeneous monolayer surface containing sorption sites that have uniform binding energy and saturation takes place when the dye molecules have filled the sites and no more adsorption can occur on that site^24^. Freundlich model assumes that the adsorption occurs on a heterogeneous surface where the binding sites are not equivalent. According to Table 1, the better fit of Langmuir model implied that the adsorption occurred on a homogeneous monolayer surface and the binding energy was uniform for every single sorption site. Additionally, the maximum adsorption capacity calculated by Langmuir model was 272 mg g^−1^, which was quite comparable to those of previously investigated activated or modified bioadsorbents^8^. The *q*_*m*,*cal*_ value was even comparable to some commercial or lab produced activated carbons^25^, which demonstrated that MSRH was a promising substitute for the widely used relatively expensive activated carbons in purifying dye-contained wastewater.

**Table 1:** Parameters of isotherm models and kinetic models in batch adsorption studies of MSRH.

Isotherm model	Langmuir model			Freundlich model				
	*q*_*m*,*cal*_ (mg g^−1^)	*K*_*L*_ (L mg^−1^)	*R* ^2^	*n*	*K*_*F*_	*R* ^2^		
	272	0.034	0.979	1.76	18.66	0.906		
**Kinetic model**	**Initial concentration (mg L** ^**−1**^ **)**	**Shaking Speeds (rpm)**	**Pseudo-second-order kinetic model**			**Intraparticle diffusion model**		
			***q***_***e***,***cal***_ **(mg g** ^**−1**^ **)**	***k***_***2***_ **(×10** ^**−3**^ **g** **mg** ^**−1**^ **min** ^**−1**^ **)**	***R*** ^***2***^	***k***_***id***_ (**mg g** ^**−1**^ **min** ^**−0**.**5**^)	***C*** (**mg g** ^**−1**^)	***R*** ^***2***^
	200	160	96	85.0	1.000	8.5	74	0.882
	300	160	145	16.2	0.999	7.9	118	0.894
	400	160	186	21.8	0.999	10.8	116	0.961
	400	60	185	9.2	0.999	19.9	151	0.942

#### Kinetic study and adsorption mechanisms by MSRH

Kinetic data were analyzed using Ho’s pseudo-second-order kinetic model and intraparticle diffusion model. Relevant parameters are presented in Table 1 and the plots are presented in Fig. 1a. Pseudo-second-order kinetic model fitted to kinetic data well, demonstrating that the rate-limiting step of this adsorption process between adsorbate (MB) and bioadsorbent (MSRH) was chemisorption. Furthermore, the *q*_*e*,*cal*_ values increased while the *k*_2_ values decreased with the increasing *C*_0_. This could be attributed to higher competition for sorption sites at higher initial dye concentrations than that at lower dye concentrations. The *k*_2_ values decreased with shaking speed, which demonstrated higher shaking speed led to faster attachment of MB molecules to the surface of bioadsorbents.

Adsorption is a multi-step process involving bulk diffusion, film diffusion, and intraparticle diffusion, thereafter, the equilibrium is achieved^6^. Particularly, the diffusion of dye molecules into the interior of the sorbent pores, i.e., intraparticle diffusion, is likely to be a rate-limiting process. Herein, intraparticle diffusion model was applied to interpret the kinetic data. According to the plots of *q*_*t*_ vs. *t* ^0.5^, the curve presented a multi-linearity correlation (i.e., three steps; see Fig. 1b). The second step is the diffusion of the adsorbate (MB molecules) from the external surface of MSRH into the pores. But the correlation coefficients of intraparticle diffusion model were between 0.882 and 0.961, indicating that the intraparticle diffusion was not the only rate controlling step; meanwhile, the linear relationship presented in Fig. 1b does not pass through the origin, indicating that some degree of boundary layer control was involved^26^.

#### Thermodynamic study

Thermodynamic studies are useful in understanding the spontaneity of the system, the dominating mechanism involved in the adsorption process, and whether the adsorption process is endothermic or exothermic. According to Table 2, the negative values of *ΔH* ^0^ confirmed the exothermic nature of the adsorption process. Meanwhile, the *ΔH* ^0^ values for all initial concentrations were lower than 40 kJ mol^−1^, suggesting that physical adsorption was the dominating mechanism^27^. The negative values of *ΔG* ^0^ indicated that the adsorption of MB onto MSRH under the present conditions was a spontaneous process. This result was similar to findings of some previous investigations^28^.

**Table 2:** Thermodynamic parameters at different initial MB concentrations.

*C*_0_ (mg L^−1^)	*ΔH* ^0^ (kJ mol^−1^)	*ΔS* ^0^ (J mol^−1^ K^−1^)	*ΔG* ^0^ (kJ mol^−1^)				*R* ^2^
			283.13 K	293.13 K	303.13 K	313.13 K	
100	−3.66	86.92	−20.95	−21.82	−22.69	−23.56	0.959
200	−3.04	68.13	−16.25	−16.93	−17.61	−18.30	0.984
300	−2.50	54.95	−13.06	−13.61	−14.16	−14.71	0.982
400	−1.82	38.88	−9.19	−9.58	−9.97	−10.36	0.992

### Comprehensive performance and mechanisms of HAPR in purifying dye wastewater

To enhance the treatment of the after-adsorption lower concentration of MB wastewater, an environmental benign bio-measure, i.e., Hybrid Adsorption-Periphyton Reactor (HAPR, Fig. S1) was constructed. As mentioned above, HAPR contains two units: the MSRH based adsorption column and the periphyton-based photo-bioreactor (PPBR).

#### Adsorption column based on MSRH

The effluent MB concentration of the adsorption column was tested at varied time intervals. Breakthrough curves, predicted by Yoon–Nelson model^29^, were applied to describe the performance of column operation (Eq. 8 in Appendix 4) and key parameters were calculated^22^.

Figure 1c showed that Yoon-Nelson model fit the experiment data well and the calculated correlation coefficients (*R* ^2^) were higher than 0.96. Additionally, when the bed depth increased, the breakthrough time also increased. The time required for 50% breakthrough (τ) obtained from the Yoon-Nelson model were 109 and 80 min for bed depth 1.5 cm and 1.0 cm, respectively. Further, the bed capacity values (per gram of bioadsorbent), q_0YN_, calculated from Eq. (9) in Appendix 4, were 343 and 379 mg g^−1^ for bed depth 1.5 cm and 1.0 cm, respectively. The data showed that a higher bed depth corresponded to a lower bed capacity value. It means MSRH was better involved in the adsorption process at lower bed depth conditions.

When the effluent dye concentration reached a certain value, which will be introduced in the following parts, the dye-loaded MSRH was removed and new MSRH was added. Meanwhile, effluent with low MB concentration (~0.5 mg L^−1^) was collected for further treatment by the following periphyton based bioreactor.

#### Prediction and collection of low concentration dye effluent by adsorption column

In order to further treat the low concentration of MB wastewater by PPBR without damaging the bioactivity of periphyton, the MB concentration of effluent collected from adsorption column should be kept at a feasible level. Consequently, it is necessary to predict the average dye concentration of the effluent after-adsorption. The average MB concentration was calculated by integrating the area below the breakthrough curve at a certain time interval (Fig. 1d, the curves were predicted by Yoon-Nelson model). For example, the average concentration before 51 min was calculated as 0.5 mg L^−1^ under the given experiment conditions (bed depth = 1.5 cm).

Figure 1e shows the average MB concentration and average accumulative MB removal percentage by adsorption column based on MSRH. The average accumulative removal percentage was very high before 51 min (reached 99.7% when bed height was 1.5 cm). But the corresponding effluent MB concentration was still visible by naked eye (at around 0.5 mg L^−1^), which indicated the need for further purification. Moreover, the collection time was responsible for MB effluent concentrations. For example, if the effluent was collected at 180 min, the average MB concentration would have reached 79 mg L^−1^. This would definitely destroy the periphyton in the following PPBR treatment. So it was important to collect effluents at predetermined time intervals. In the present study, 0.2 and 0.5 mg L^−1^ MB effluent solutions of the adsorption column were collected and added to the following PPBR unit.

#### MB removal performance of PPBR

As mentioned above, PPBR was designed to further treat the lower concentrations of MB solution. In order to (1) simulate the discharge content of actual wastewater and (2) keep the continuous treatment of this dye-contaminated wastewater without damaging the periphyton in PPBR, the average MB concentrations of the effluents were set at around 0.5 and 0.2 mg L^−1^ (as mentioned above), and were added to the PPBR process every 48 h.

**Figure 2:**
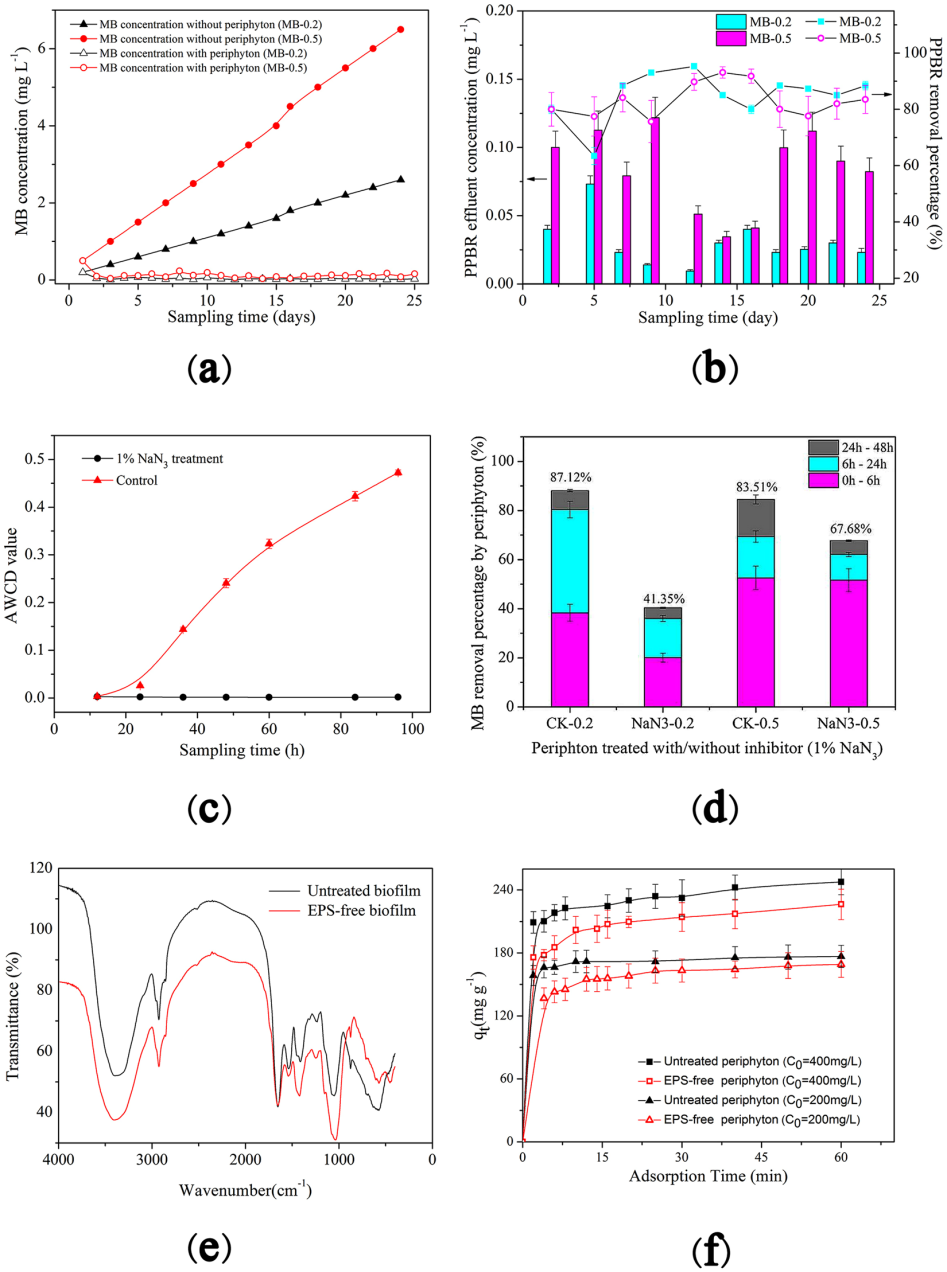
**(a)** Dye concentration without periphyton (the theoretical dye accumulated) and dye concentration with periphyton in PPBR (MB was added every 2 days, lasted around 4 weeks); **(b)** Effluent MB concentration and removal percentages of PPBR (MB was added every 2 days and the effluents were collected and texted before the next addition of MB wastewater); **(c)** AWCD value of periphyton with/without inhibition (microbial activity was determined by Biolog^TM^ ECO Microplates); **(d)** Removal performance by periphyton treated with/without NaN_3_ in PPBR (NaN_3_ concentration = 1%, w/v); **(e)** FTIR spectra of untreated periphyton and EPS-free periphyton, and **(f)** The adsorption dynamics of MB by untreated periphyton and EPS-free periphyton.

Figure 2a showed that PPBR process was observed an obvious dye-removing ability. Although low concentration of MB was added to PPBR every 48 h, the effluent was dramatically purified during the sampling time. According to Fig. 2b, around 80–85% of the initial MB concentration was removed in 48 h.

For 0.5 mg L^−1^ and 0.2 mg L^−1^ treatment, the average MB removal percentages during the test period were 83.2% and 84.9%, respectively. The corresponding average MB concentrations were 0.08 and 0.03 mg L^−1^, respectively. These concentrations of effluents are invisible to naked eyes. The results showed that HAPR process successfully purified the high-level dye-contaminated wastewater and the treatment of lower initial dye concentration (0.2 mg L^−1^) resulted in a higher removal percentage (84.9%). This may be attributed to the important role of adsorption process that played during the PPBR purification process.

#### Proposed mechanism of MB removal by periphyton in PPBR

To understand the removal mechanisms of MB by HAPR, the mechanisms involved both in adsorption column and PPBR should be investigated. The adsorption mechanisms of MSRH have been discussed in the batch study in section 3.1, consequently, the removal mechanisms of MB by PPBR will be emphasized in this part. Obviously, periphyton played a key role in purifying MB-contained wastewater by PPBR. Herein, the purification mechanisms of MB by periphyton will be investigated.

The removal of hazardous materials by living organisms, such as periphyton, can take place by both biodegradation and physical/chemical mechanisms such as adsorption^11^. Herein, we planned to investigate the following two questions: (1) whether biodegradation was involved in the MB removal, and (2) if biodegradation was involved, what were the specific contributions of adsorption and biodegradation.

It has been reported that NaN_3_ can suppress microbial respiration and inhibit the process of biodegradation^18^. As mentioned in section 2.7, if the microbial activities of periphyton were completely inhibited by NaN_3_, the removal of MB under this condition was mainly due to adsorption process; on the contrary, if the periphyton was not inhibited, the removal of MB may be both due to adsorption and biodegradation. Through applying NaN_3_, the contribution of bioadsorption and biodegradation of MB by periphyton can be assessed. During this process, it was necessary to confirm whether the 1% (w/v) NaN_3_ completely inhibited periphyton under the present experiment conditions. Hence, the AWCD values obtained through Biolog^TM^ ECO Microplates are presented in Fig. 2c. According to this figure, the AWCD values of NaN_3_-inhibited periphyton were close to 0, which demonstrated the microbial respiration of the NaN_3_-treated periphyton was completely inhibited.

Figure 2d showed the MB removal percentages by NaN_3_-inhibited periphyton and non-inhibited periphyton in PPBR. It was clear that the NaN_3_-inhibited periphyton had a clear inferior performance. The MB removal percentage (with the initial MB concentration 0.2 mg L^−1^) was 87% and 41% for control and NaN_3_-inhibited periphyton, respectively. It means, from the total amount of MB (i.e., 87% of the total MB concentration) removed in 48 hours, 41% was due to physical-chemical processes (i.e., adsorption) and 46% was due to biological process (i.e., biodegradation). Additionally, for the NaN_3_-inhibited periphyton, a larger amount of MB was removed during the first 6 h. The fast removal was similar to that observed in the adsorption alone process, which achieved equilibrium in a short time (usually from minutes to hours)^30^. This also indicated that the removal of MB was first dominated by adsorption at the fast removal stage (around 6 hours) and then the adsorption reached saturation, thereafter, biodegradation played a key role.

At higher initial MB concentration (0.5 mg L^−1^), the trend was similar to that of 0.2 mg L^−1^ treatment, but adsorption played a larger part (68% was due to adsorption and 16% was due to biodegradation). That may be due to the following two reasons: (1) the high adsorption capacity of extracellular polymeric substances^31^ on the surface of periphyton, and (2) the adsorption process was much faster than the biodegradation process.

The extracellular polymeric substances (EPS) can reach 98% of the total organic carbon fraction of microbial aggregates, such as periphyton^32^. EPS usually contain carbohydrates and proteins^11^, which provide a large amount of functional groups, such as –COOH and –OH. Herein, in order to identify the functional groups associated with the adsorption process, FTIR spectra of the untreated and EPS-free periphytons were measured since each functional group possesses a unique energy absorption band. This can offer more useful information about periphyton structure. The preparation of EPS-free periphyton followed a previous report^33^. The spectra are illustrated in Fig. 2e. It could be noted that the peak near 1643 cm^−1^ corresponds to C=O stretching in amide group, and those at 1551 cm^−1^ and 1541 cm^−1^ were attributable to N–H bending and C–N stretching in amide group, respectively^34^. Peaks at 1417 cm^−1^ and 1396 cm^−1^ were mainly assigned to C–O symmetric stretching of carboxyl groups, while peaks at 1047 cm^−1^ and 1036 cm^−1^ were associated with P=O stretching of phosphate groups^35, 36^. Several functional groups such as amino, phosphate and carboxyl were found on the EPS-free periphyton surface. It also indicated the removal of EPS from periphyton surface made no significant difference on functional groups for their similarity in peak positions. This result was in line with previous report that the FTIR spectra of untreated and EPS-free *Bacillus subtilis* and *Pseudomonas putida* are similar^37^. The presence of various functional groups may provide a large adsorption capacity of EPS for hazardous materials. Herein, an adsorption experiment of MB by periphyton and EPS-free periphyton was conducted, and Fig. 2f shows the adsorption process. Based on this figure, the adsorption capacity of untreated periphyton was higher than that of EPS-free periphyton. According to Langmuir model (data not shown), the adsorption capacity of untreated periphyton was around 30 mg g^−1^ higher than that of EPS-free periphyton. This experiment confirmed the important role of EPS in the adsorption process of MB by periphyton in PPBR.

**Figure 3:**
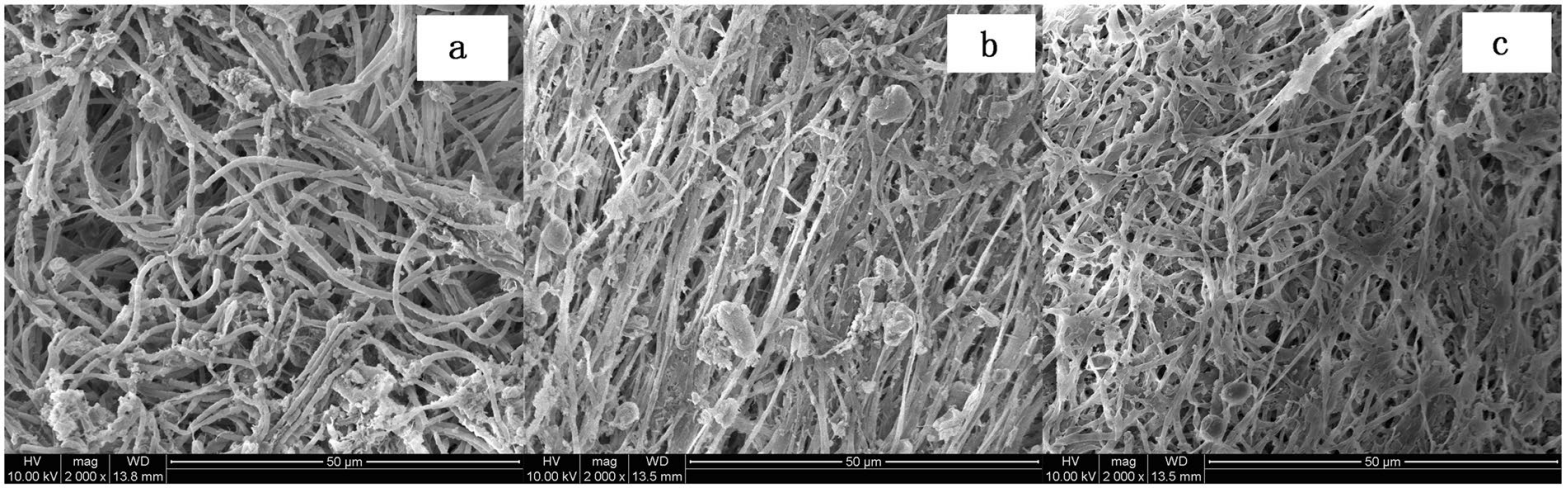
ESEM images of **(a)** periphyton without MB (2000×); **(b)** periphyton with 0.2 mg L^−1^ MB (2000×), and (**c**) periphyton with 0.5 mg L^−1^ MB (2000×).

ESEM micrographs presented in Fig. 3a revealed that periphyton was filamentous fungi and algae. However, images presented in Fig. 3b and c indicated that periphyton turned to be a net structure and appeared to be tighter than the untreated periphyton in Fig. 3a. This demonstrated MB had apparently exerted an influence on the texture of periphyton. Previous studies had been reported that pollutants such as Cd, Cr and Pb could reduce growth of periphyton communities^38^ and external injuries were caused by the toxicity of the Azo Dye Methyl Red^39^. To some extent, these toxic pollutants all could lead to the change of structure in periphyton and the changes in turn might be a direct response of defense mechanism by periphyton.

Moreover, the presence of EPS may also provide a buffering zone and keep the enclosed living organisms from fatal damage caused by these hazardous materials. This was important for the following potential biodegradation of MB by living organisms in periphyton.

As mentioned above, biodegradation was another important mechanism to reduce dye concentration from water. Biodegradation was performed when microorganisms alter the original structure of dyes and completely mineralize them to water and carbon dioxide. It has been reported that microalgae could remove dyes because of their quickly response to environmental changes due to their fast cell proliferation compared to higher plants^3^. The existence of various algae in periphyton will contribute to the biodegradation of MB in the present study. Photodegradation could also play a part in dye removal, but under the present conditions, it would not be a dominant mechanism because (1) adsorption process will take much less time than photodegradation; (2) periphyton contains many algae species, EPS, minerals, which can effectively block the light from outside; and (3) sunlight alone (i.e., in the absence of catalysts) usually exhibits a low efficiency in degrading contaminants. Additionally, a shade net was applied to reduce the effect from sunlight photodegradation during the experiment period. Experiments also confirmed the inefficiency of photodegradation in the present study (data not shown).

**Figure 4:**
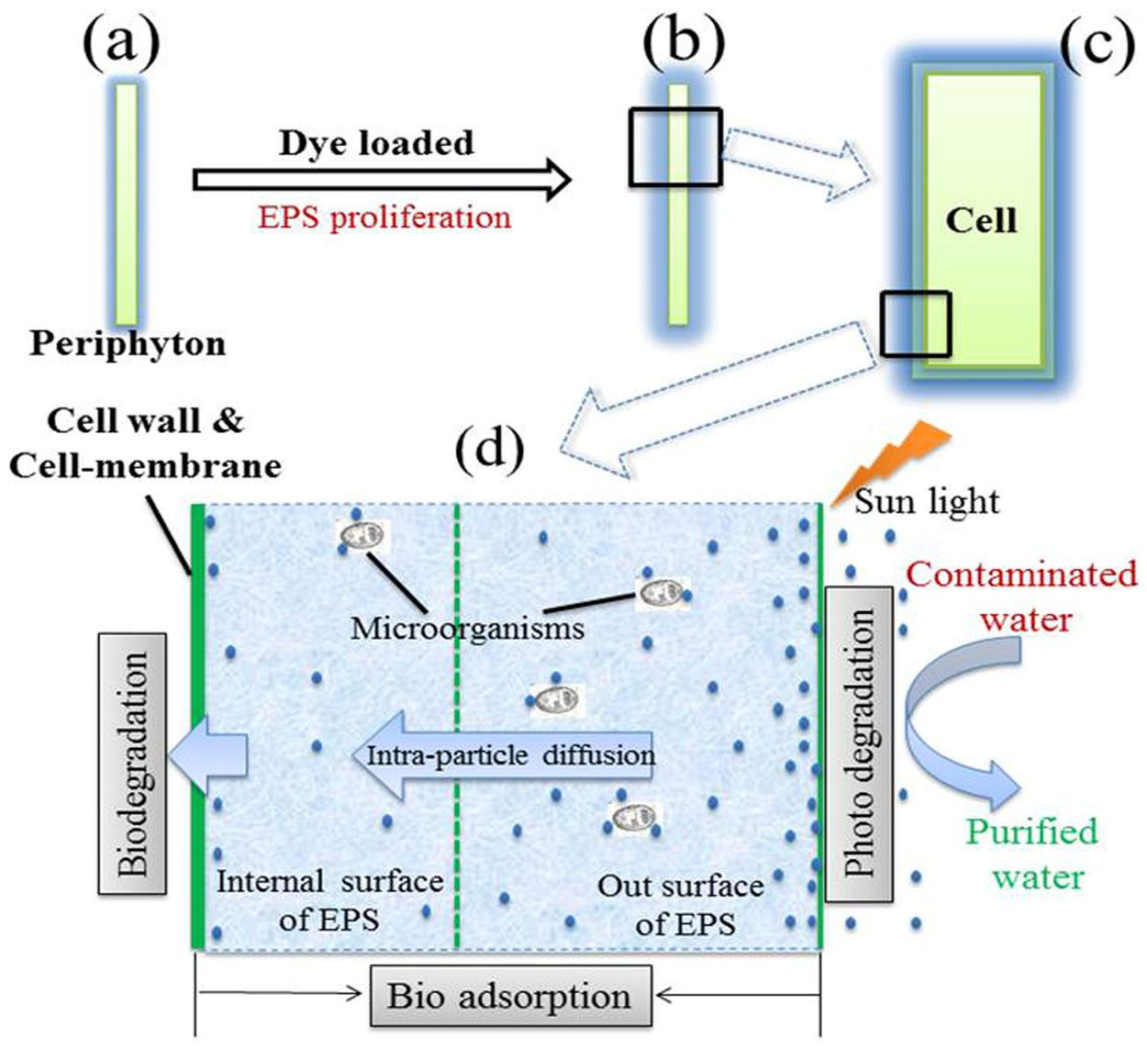
Schematic illustration for dye removal mechanisms by periphyton.

In conclusion, the removal mechanism of MB by periphyton can be illuminated by Fig. 4. Firstly, MB molecules were adsorbed on the outer surface of periphyton; then through intra-particle diffusion MB molecules penetrated EPS to bind with functional groups on the internal surface of the EPS/Cell wall. After that, some of MB molecules were mineralize by algae cell or other microorganisms. At the same time, EPS present on the surface of cells get expanded and to bind more MB molecules, which will lower the environmental stress caused by the existence of MB. Photodegradation was not the dominant mechanism in this process.

## Conclusion

This study developed a hybrid adsorption-periphyton reactor (HAPR), which included an adsorption column (the first stage) and a periphyton-based photo-bioreactor (PPBR, the second stage). Herein, periphyton was for the first time applied as a bio-medium and periphyton-based photo-bioreactor (PPBR) was built to treat dye contaminated wastewater. Results showed that HAPR was a promising environmental-benign bio-system, and with continuous addition of MB solution, the effluent MB concentration of HAPR was lower than 0.1 mg L^−1^ under the present experiment conditions. The dominant removal mechanisms of MB by periphyton were bioadsorption and biodegradarion process, while EPS played an important role in the adsorption process.

## Electronic supplementary material

Supporting information

## References

[1] Shabbir S, Faheem M, Ali N, Kerr PG, Wu Y (2017). Evaluating role of immobilized periphyton in bioremediation of azo dye amaranth. Bioresource Technology.

[2] Feng Y (2013). Adsorption of dyestuff from aqueous solutions through oxalic acid-modified swede rape straw: adsorption process and disposal methodology of depleted bioadsorbents. Bioresource Technology.

[3] Hernández-Zamora M (2015). Bioremoval of the azo dye Congo Red by the microalga Chlorella vulgaris. Environmental Science and Pollution Research.

[4] Sewu DD, Boakye P, Woo SH (2017). Highly efficient adsorption of cationic dye by biochar produced with Korean cabbage waste. Bioresource Technology.

[5] Chang Y, Lai J-Y, Lee D-J (2016). Thermodynamic parameters for adsorption equilibrium of heavy metals and dyes from wastewaters: Research updated. Bioresource Technology.

[6] Feng Y (2012). Methylene blue adsorption onto swede rape straw (*Brassica napus* L.) modified by tartaric acid: equilibrium, kinetic and adsorption mechanisms. Bioresource Technology.

[7] Tan X-f (2017). Biochar as potential sustainable precursors for activated carbon production: Multiple applications in environmental protection and energy storage. Bioresource Technology.

[8] Ahmed MJ (2016). Application of agricultural based activated carbons by microwave and conventional activations for basic dye adsorption: Review. Journal of Environmental Chemical Engineering.

[9] Yu J-x (2012). A situ co-precipitation method to prepare magnetic PMDA modified sugarcane bagasse and its application for competitive adsorption of methylene blue and basic magenta. Bioresource Technology.

[10] Waghmode TR, Kurade MB, Kagalkar AN, Govindwar SP (2012). Differential fate of metabolism of a disperse dye by microorganisms *Galactomyces geotrichum* and *Brevibacillus laterosporus* and their consortium GG-BL. Journal of Environmental Sciences.

[11] Wu Y, Li T, Yang L (2012). Mechanisms of removing pollutants from aqueous solutions by microorganisms and their aggregates: A review. Bioresource Technology.

[12] Shabbir S, Faheem M, Ali N, Kerr PG, Wu Y (2017). Periphyton biofilms: A novel and natural biological system for the effective removal of sulphonated azo dye methyl orange by synergistic mechanism. Chemosphere.

[13] Yang H-Y (2016). Process and kinetics of azo dye decolourization in bioelectrochemical systems: effect of several key factors. Scientific Reports.

[14] Lu H, Yang L, Shabbir S, Wu Y (2014). The adsorption process during inorganic phosphorus removal by cultured periphyton. Environmental Science and Pollution Research.

[15] Battin TJ, Besemer K, Bengtsson MM, Romani AM, Packmann AI (2016). The ecology and biogeochemistry of stream biofilms. Nat Rev Micro.

[16] Li G (2016). Temporal Succession of Ancient Phytoplankton Community in Qinghai Lake and Implication for Paleo-environmental Change. Scientific Reports.

[17] Quero GM, Fasolato L, Vignaroli C, Luna GM (2015). Understanding the association of Escherichia coli with diverse macroalgae in the lagoon of Venice. Scientific Reports.

[18] Wu Y, He J, Yang L (2010). Evaluating adsorption and biodegradation mechanisms during the removal of Microcystin-RR by periphyton. Environmental Science & Technology.

[19] Miao L (2015). Effects of pH and natural organic matter (NOM) on the adsorptive removal of CuO nanoparticles by periphyton. Environmental Science and Pollution Research.

[20] Inaba T, Hori T, Aizawa H, Ogata A, Habe H (2017). Architecture, component, and microbiome of biofilm involved in the fouling of membrane bioreactors. npj Biofilms and Microbiomes.

[21] Li X, Jin X, Zhao N, Angelidaki I, Zhang Y (2017). Novel bio-electro-Fenton technology for azo dye wastewater treatment using microbial reverse-electrodialysis electrolysis cell. Bioresource Technology.

[22] Ponnusami V, Vikram S, Srivastava SN (2008). Guava (Psidium guajava) leaf powder: Novel adsorbent for removal of methylene blue from aqueous solutions. Journal of Hazardous Materials.

[23] Balser TC, Wixon DL (2009). Investigating biological control over soil carbon temperature sensitivity. Global Change Biology.

[24] Mahmoud DK, Salleh MAM, Karim WAWA, Idris A, Abidin ZZ (2012). Batch adsorption of basic dye using acid treated kenaf fibre char: Equilibrium, kinetic and thermodynamic studies. Chemical Engineering Journal.

[25] Marrakchi F, Ahmed MJ, Khanday WA, Asif M, Hameed BH (2017). Mesoporous-activated carbon prepared from chitosan flakes via single-step sodium hydroxide activation for the adsorption of methylene blue. International Journal of Biological Macromolecules.

[26] Tang H, Zhou W, Zhang L (2012). Adsorption isotherms and kinetics studies of malachite green on chitin hydrogels. Journal of Hazardous Materials.

[27] Lin X (2017). Adsorption behavior of levulinic acid onto microporous hyper-cross-linked polymers in aqueous solution: Equilibrium, thermodynamic, kinetic simulation and fixed-bed column studies. Chemosphere.

[28] Gupta VK, Gupta B, Rastogi A, Agarwal S, Nayak A (2011). Pesticides removal from waste water by activated carbon prepared from waste rubber tire. Water Research.

[29] Ghali AE, Baouab MHV, Roudesli MS (2011). Preparation, characterization and application of a [copper (II)/ethylenediamine–cotton] complex for the removal of AB25 from aqueous solution in a laboratory scale column. Chemical Engineering Journal.

[30] Liu J, Li E, You X, Hu C, Huang Q (2016). Adsorption of methylene blue on an agro-waste oiltea shell with and without fungal treatment. Scientific Reports.

[31] Wei L (2017). Adsorption of Cu^2+^ and Zn^2+^ by extracellular polymeric substances (EPS) in different sludges: Effect of EPS fractional polarity on binding mechanism. Journal of Hazardous Materials.

[32] Ras M, Lefebvre D, Derlon N, Paul E, Girbal-Neuhauser E (2011). Extracellular polymeric substances diversity of biofilms grown under contrasted environmental conditions. Water Research.

[33] Ellwood NT, Di Pippo F, Albertano P (2012). Phosphatase activities of cultured phototrophic biofilms. Water research.

[34] Dogsa I, Kriechbaum M, Stopar D, Laggner P (2005). Structure of bacterial extracellular polymeric substances at different pH values as determined by SAXS. Biophysical journal.

[35] Ueshima M, Ginn BR, Haack EA, Szymanowski JES, Fein JB (2008). Cd adsorption onto Pseudomonas putida in the presence and absence of extracellular polymeric substances. Geochimica et Cosmochimica Acta.

[36] Lorite GS (2011). The role of conditioning film formation and surface chemical changes on Xylella fastidiosa adhesion and biofilm evolution. J Colloid Interf Sci.

[37] Fang L (2011). Role of extracellular polymeric substances in Cu(II) adsorption on Bacillus subtilis and Pseudomonas putida. Bioresource technology.

[38] Bere T, Chia MA, Tundisi JG (2012). Effects of Cr III and Pb on the bioaccumulation and toxicity of Cd in tropical periphyton communities: Implications of pulsed metal exposures. Environ Pollut.

[39] Sharma S, Sharma S, Pathak S, Sharma KP (2003). Toxicity of the azo dye methyl red to the organisms in microcosms, with special reference to the guppy (Poecilia reticulata Peters). Bulletin of environmental contamination and toxicology.

