# Supplementary material for: Purification of Dye-stuff Contained Wastewater by a Hybrid Adsorption-Periphyton Reactor (HAPR): Performance and Mechanisms
Source: Sci Rep. 2017 Aug 29;7:9635. doi: 10.1038/s41598-017-10255-8 (PMC5574932; doi:10.1038/s41598-017-10255-8)
Supplement: Supplementary file 1 — Supporting information [file 41598_2017_10255_MOESM1_ESM.doc]

## Purification of Dye-stuff Contained Wastewater by a Hybrid Adsorption-Periphyton Reactor (HAPR): Performance and Mechanisms

**Yanfang Feng^1,2,4^; Lihong Xue^1^;** **Jingjing Duan^1^; Dionysios D. Dionysiou^5^; Yudong Chen^3^*; Linzhang Yang^1,2^*, Zhi Guo^1.^**

^1^ Institute of Agricultural Resources and Environment, Jiangsu Academy of Agricultural Sciences, Nanjing 210014, China

^2^ State Key Laboratory of Soil and Sustainable Agriculture, Institute of Soil Science, Chinese Academy of Sciences, Nanjing 210008, China

^3^ Nanjing Institute of Environmental Science, MEP, Nanjing, 210042, China

^4^ Stockbridge School of Agriculture, UMASS, MA 01003, USA

^5^ Environmental Engineering and Science Program, University of Cincinnati, Cincinnati, OH 45221-0071, USA

^*^Corresponding author:

Prof. Dr. Linzhang Yang, Dr. Yudong Chen

Tel (Fax): +86-25-84391526

Address: No.50 Zhongling Rd., Nanjing, Jiangsu, 210014, China

E-mail: [jaasfengyanfang@163.com](mailto:jaasfengyanfang@163.com) (Y. Feng); [lzyang@issas.ac.cn](mailto:lzyang@issas.ac.cn) (L. Yang); [cyd@nies.org](mailto:cyd@nies.org) (Y. Chen)

Catalogue

[Appendix 1. Schematic diagram of the Hybrid Adsorption-Periphyton Reactor 3](#__RefHeading___Toc485554176)

[Appendix 2. Column adsorption study 4](#__RefHeading___Toc485554177)

[Appendix 3. Periphyton-based photo bioreactor 5](#__RefHeading___Toc485554178)

[Appendix 4. Mathematical models of adsorption process 6](#__RefHeading___Toc485554179)

[Appendix 5. Biolog experiment 7](#__RefHeading___Toc485554180)

[Appendix 6. Characterization of bioadsorbent (MSRH) 8](#__RefHeading___Toc485554181)

[Appendix 7. Effect of operating conditions on adsorption 11](#__RefHeading___Toc485554182)

[Appendix 8. Confocal microscopy (CLSM) to observe periphyton in PPBR 14](#__RefHeading___Toc485554183)

[Appendix 9. Nomenclature 16](#__RefHeading___Toc485554184)

[Reference 16](#__RefHeading___Toc485554185)

## Appendix 1. Schematic diagram of the Hybrid Adsorption-Periphyton Reactor


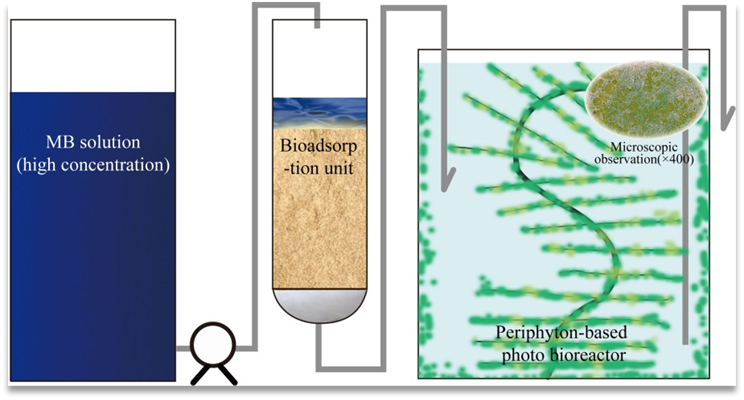


**Fig. 1S** Schematic diagram of the Hybrid Adsorption-Periphyton Reactor (HAPR).

## Appendix 2. Column adsorption study

The adsorption column is schematically present below. Specifically, to avoid the leakage of bioadsorbent particles, some glass wool was placed at the column bottom. The schematic diagram of the adsorption column is presented in the following figure.


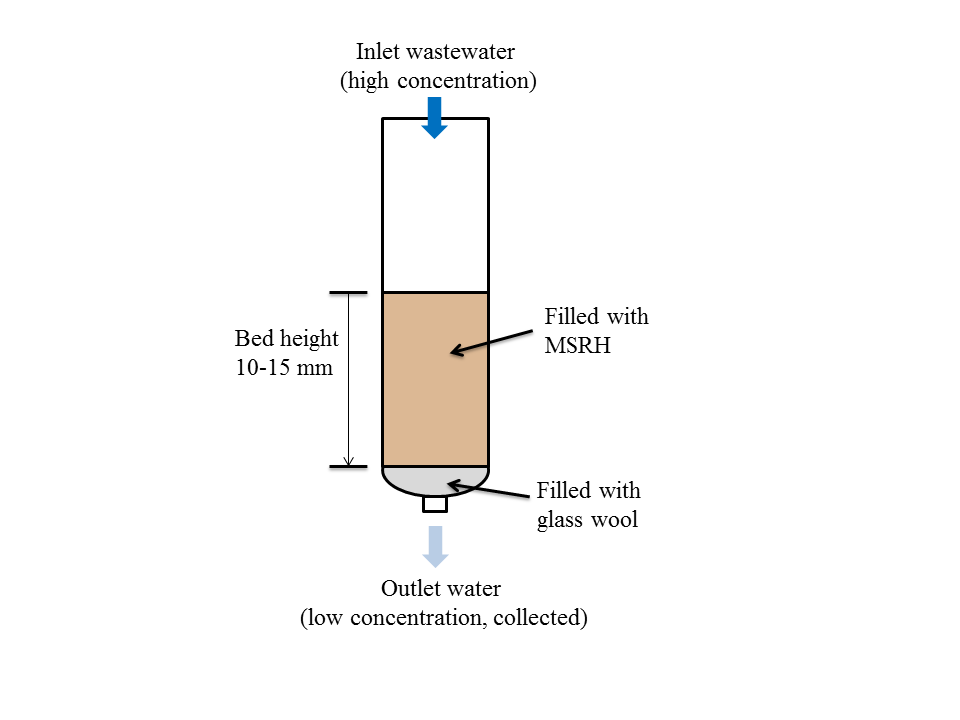


**Fig. 2S** The schematic diagram of the adsorption column.

## Appendix 3. Periphyton-based photo bioreactor


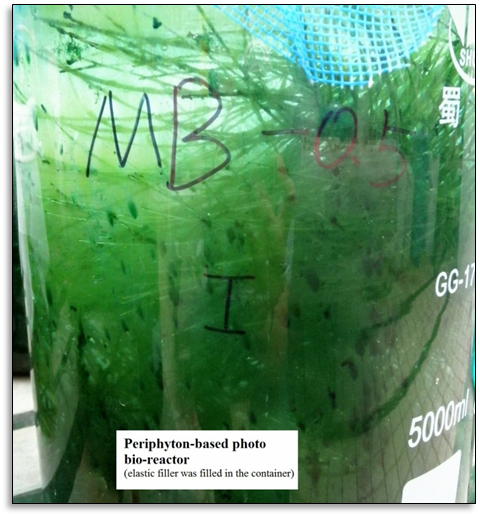


**(a)**


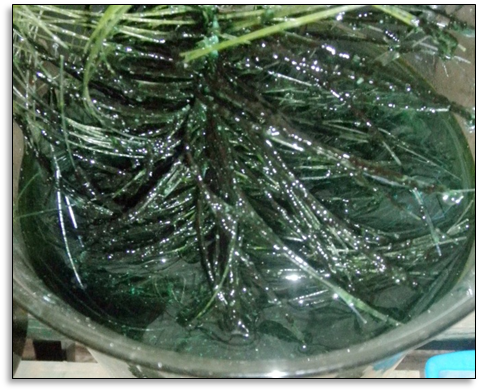


**(b)**

**Fig. 3S** The picture of the periphyton-based photo bioreactor.

## Appendix 4. Mathematical models of adsorption process

**Table 1S** Mathematical models of adsorption process.

| Objectives | Mathematical models  & target parameters | Equations | Equation  No. |
| --- | --- | --- | --- |
| Equilibrium isotherms | Langmuir model | $\frac{C_{\text{e}}}{q_{e}}=\frac{1}{q_{m}}C_{e}+\frac{1}{K_{L}q_{m}}$ | (1) |
|  | Freundlich model | $\ln q_{e}=\ln K_{F}+\frac{1}{n}\ln C_{e}$ | (2) |
| Kinetic study | Pseudo-first-order kinetic model | $\log(q_{e}-q_{t})=\log q_{e}-\frac{k_{1}}{2.303}t$ | (3) |
|  | Ho’s pseudo-second-order kinetic model | $\frac{t}{q_{t}}=\frac{1}{k_{2}q_{e}^{2}}+\frac{t}{q_{e}}$ | (4) |
| Adsorption mechanisms | Intra-particle diffusion model | $q_{t}=k_{id}t^{0.5}+C$ | (5) |
| Thermodynamic study | ---- | $\Delta G^{0}=\Delta H^{0}-T\Delta S^{0}$ | (6) |
|  |  | $\log(\frac{q_{e}}{C_{e}})=\frac{\Delta S^{0}}{2.303R}+\frac{-\Delta H^{0}}{2.303RT}$ | (7) |
| Column study | Yoon-Nelson model | $\ln(\frac{C_{t}}{C_{0}-C_{t}})=K_{YN}-\tau K_{YN}$ | (8) |
|  |  | $q_{0YN}=\frac{C_{0}Q\tau}{1000m}$ | (9) |

## Appendix 5. Biolog experiment

150 μL prediluted periphyton-water mixture was added into each well of every Biolog^TM^ ECO Microplate, and was incubated at 25 ^o^C. The absorbance was determined at 590 nm using a Biolog Microplate Reader at predetermined time intervals for several days.

The method to calculate AWCD value is as follows:

$$AWCD=\frac{1}{31}\sum_{i-1}^{31} (R_{it}-R_{0t})$$

$$R_{si}=\frac{R_{i}-R_{0}}{\sqrt{\frac{1}{31}\sum_{i-1}^{31} (R_{i}-AWCD)^{2}}}$$

Where R_it_ and R_0t_ are the absorbance values of the sole carbon source I and the water blank at time t, respectively, and R_si_ is the standardized absorbance values.

Shannon indices are calculated as follows:

$$H'=-\sum p_{i}\ln(p_{i})$$

where *p*_i_ is the proportional color development of the i^th^ well relative to the total color development of all plate wells.

## Appendix 6. Characterization of bioadsorbent (MSRH)

#### SEM analysis

Scanning electron microscope was used to observe the surface morphology of MSRH and MB loaded MSRH (Appendix 4). MSRH exhibited a rough surface with plenty of caves or holes, which would lead to a comparatively large surface. The structure was mainly due to the remains of cell wall and microwave assisted activation. Further, the SEM image of dye-loaded MSRH (MSRH-MB) showed an obviously changed surface morphology: the surface became much smoother than that of MSRH.


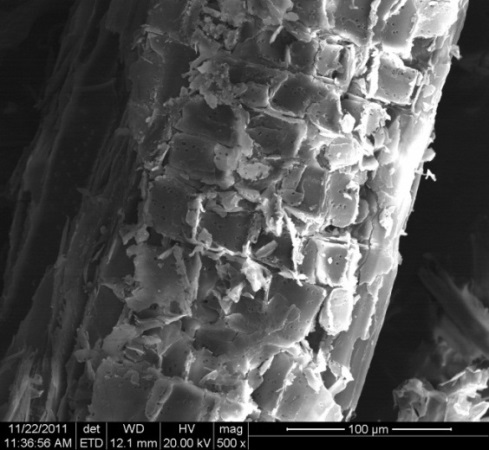

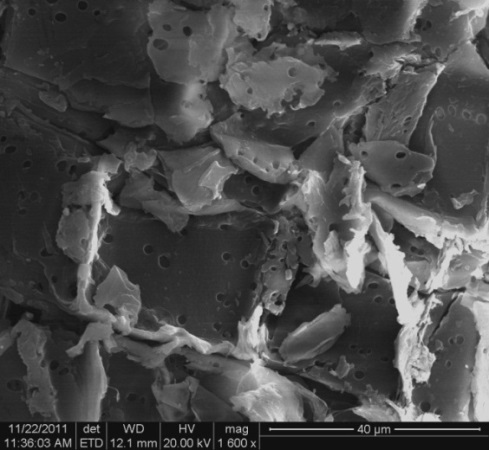


**(a)**  **(b)**


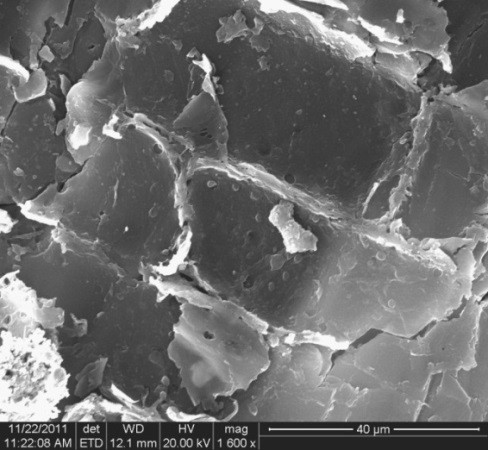


**(c)**

**Fig. 4S** SEM images of (a) MSRH (×500); (b) MSRH (×1600) and (c) methylene blue loaded MSRH-MB (×1600).

#### FTIR study

FTIR study (Fig. 5S and Table 2S) showed that some functional groups on the sorbent surface were involved in the adsorption process. Specifically, the obviously changed peaks at around 3410, 2923, 1734 and 1607 cm^-1^ proved that functional groups such as –OH, -COOH, C=O and C=C, played an important role in the adsorption process. Moreover, the newly emerged peak after adsorption, such as peak at 682 cm^-1^, proved the presence of MB molecules on the surface of MSRH-MB.


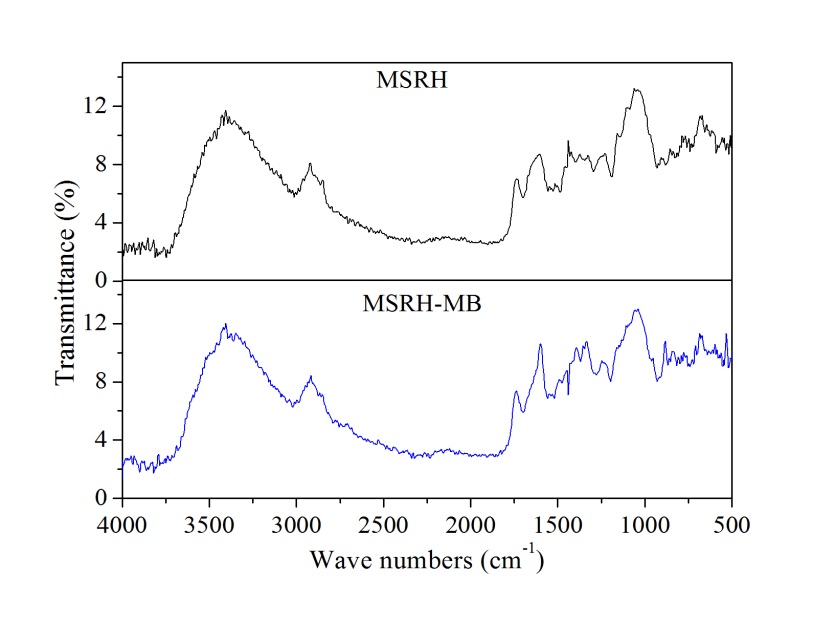


**Fig. 5S** FTIR spectra of MSRH that before and after adsorption of MB.

**Table 2S Peak changes of FTIR spectral and relative functional groups of MSRH and MB-attached MSRH.**

| Before adsorption  (cm^-1^) | After adsorption  (cm^-1^) | Peak changes  (cm^-1^) | Relative functional groups |
| --- | --- | --- | --- |
| 3410.06 | 3403.46 | -6.6 | -O-H |
| 2923.58 | 2916.98 | -6.6 | -COOH |
| 1734.91 | 1734.91 | More sharp | C=O |
| 1607.23 | 1593.29 | -13.94 | C=C |
| 1431.13 | 1445.05 | -13.92 | C-N (amide) |
| 1336.43 | 1336.48 | 0.05 | C-N (amine) |
| 1046.65 | 1039.31 | -7.34 | R-CH=CH_2_ |
| none | 681.97 | Newly emerged | Ar-R (C-H bond) |

#### Particle size distribution

According to Fig 6S, the volume mean diameter of MSRH was 140 μm, which was slightly lower than the sieve pore size.


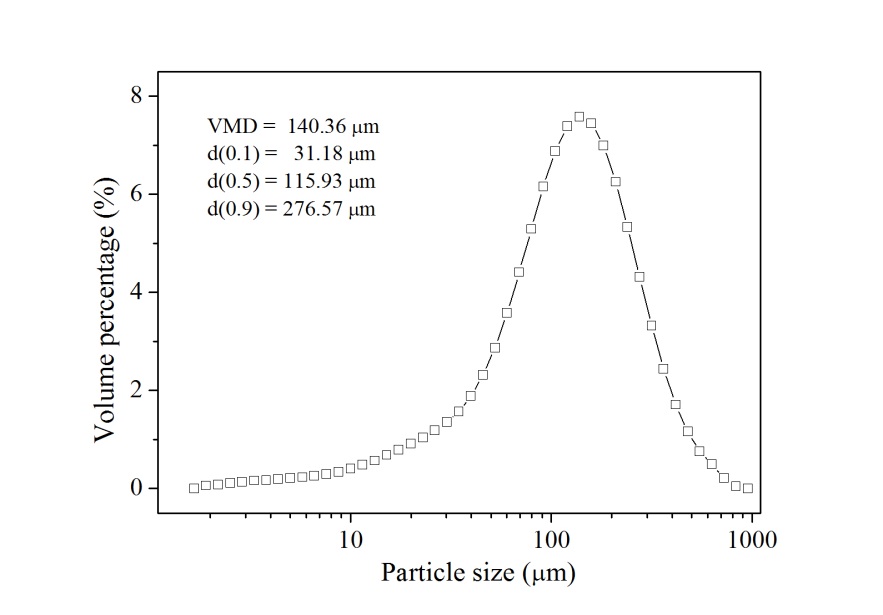


**Fig. 6S** Particle size distribution of MSRH

## Appendix 7. Effect of operating conditions on adsorption

#### Effect of sampling time and initial MB concentrations

According to Fig. 7S-a, *q_t_* values increased with the sampling time before the equilibrium was attained. Additionally, *q_t_* values were higher at higher initial MB concentrations than that of lower initial MB concentrations. The driving force of the adsorption process, which was mainly due to the dye concentration gradient between solution and solid surface, increased with increase in initial dye concentration. Consequently, q_t_ values would be higher in case of higher initial MB concentrations.

Moreover, according to Fig. 7S-a, equilibrium time at higher initial dye concentrations (~60 min) was longer than that of lower initial dye concentrations (~10 min). At lower initial dye concentrations, the sorption sites were sufficient to attach the major part of MB molecules in the solutions. This process was very fast. But at higher initial dye concentrations, a large part of the unattached dye molecules need to penetrate the surface boundary layer and enter into the caves & holes of the sorbent by intraparticle diffusion. Consequently, the equilibrium time at higher initial dye concentrations was much longer than that at lower initial dye concentrations because of the rate-limiting process (intraparticle diffusion).

#### Effect of sorbent dose

According to 7S-b, when the dose increased, the q_e_ values decreased and the removal percentages increased. That because higher dose led to a higher driving force (or higher concentration gradient between sorbent surface and the solution) of the adsorption process.

#### Effect of solution pH

In this study, the effect of pH was examined on MB adsorption in the MSRH process. According to Fig. 7S-c, the removal percentage (R %) of MB was much lower at pH 2.74 than that at pH 3.14. But when the solution pH values increased thereafter, the R % values did not change much. At highly acidic conditions (pH ≤ 3.14), the number of positively charged sorption sites increased, and due to the electrostatic repulsion, the sorption of MB^+^ by this bioadsorbent decreased accordingly.

#### Effect of role of ionic strength

Because the actual colored effluents usually contain various salts, consequently, in this study, three common salts were selected to investigate their effects on MB adsorption by MSRH. According to Fig. 7S-d, the decrease of removal percentage was obvious when the salt concentrations increased from 0.005 to 0.200 mol L^-1^. This phenomenon indicated that the competitive effect of cationic ions was an important interference to the adsorption process, and if MSRH would be used in the future, the negative effects of the presence of salts should be taken into consideration. Meanwhile, the negative effect of CaCl_2_ and MgCl_2_ were more prominent than that of NaCl. It could be attributed to the larger hydrated radius and more e^+^ (positive charge) of Ca^2+^ and Mg^2+^ than that of Na^+^.

#### Effect of shaking speed on adsorption process

According to Fig. 7S-e, the shaking speed has an obvious effect on the adsorption rate and the amount of MB uptake. The larger q_t_ values and faster initial sorption rate at higher shaking speed can be attributed to the increased system mobility and decreased film resistance to mass transfer. Additionally, the boundary layer diffusion effects increased when the shaking speed decreased.


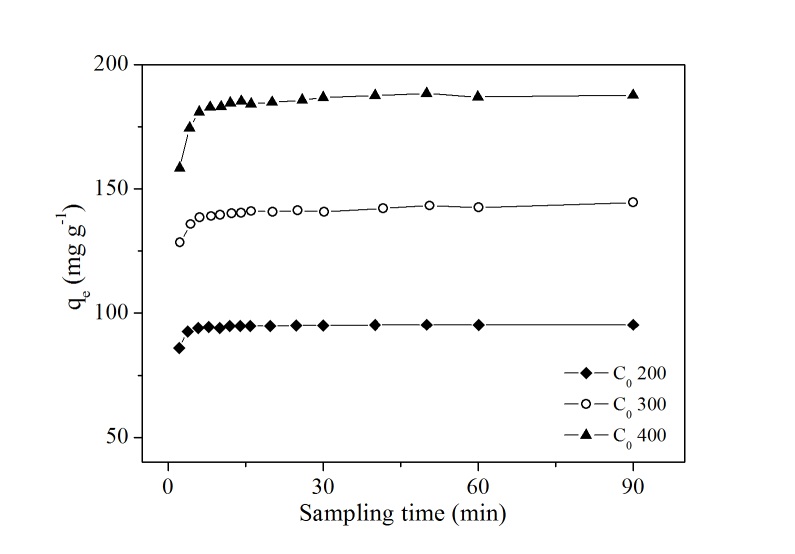

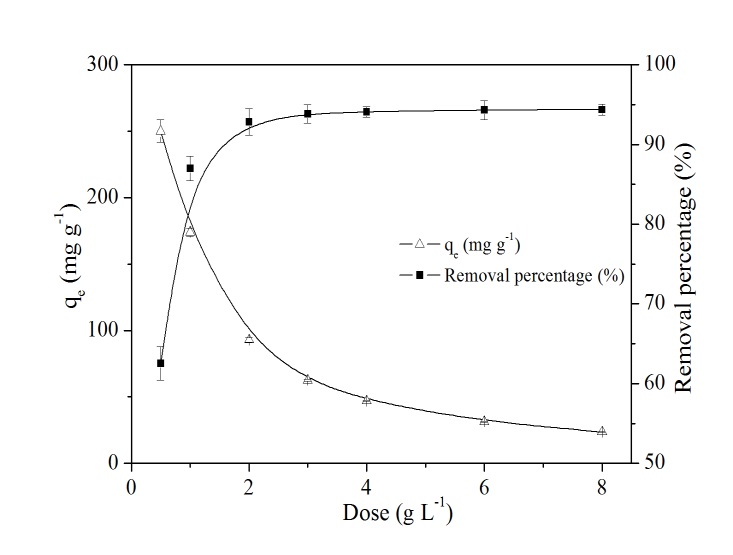


1. **(b)**


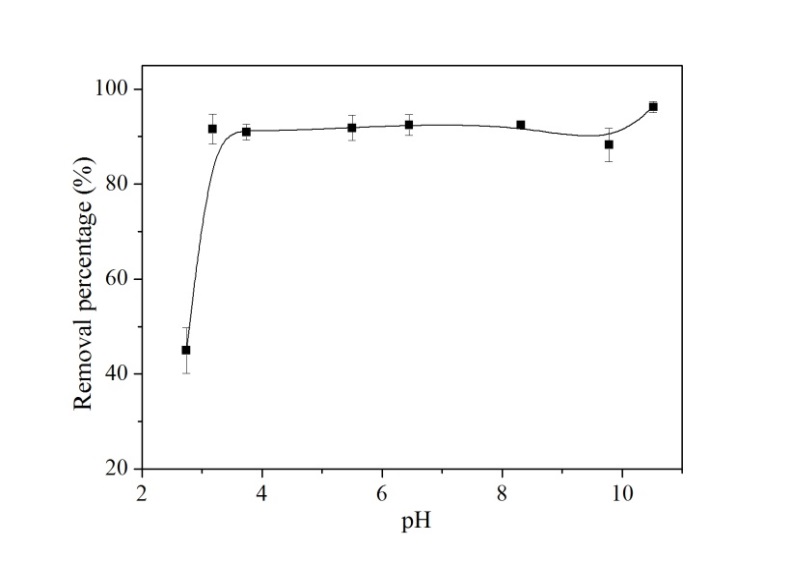

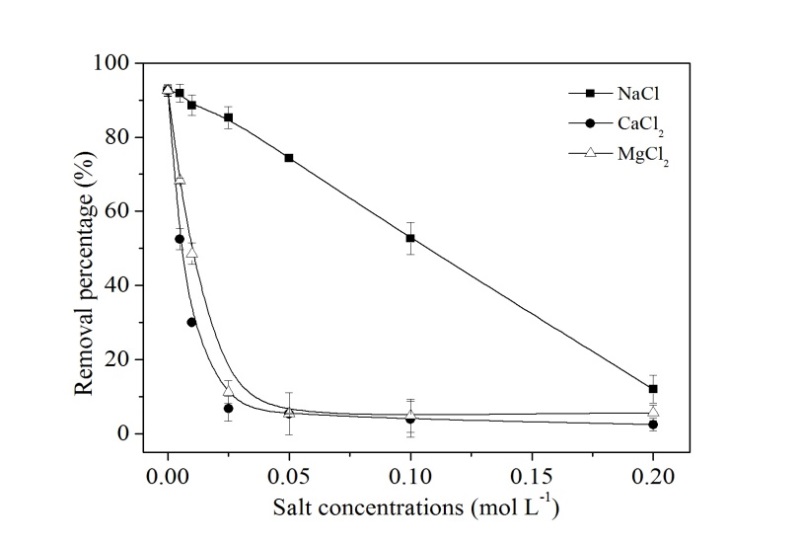


1. **(d)**


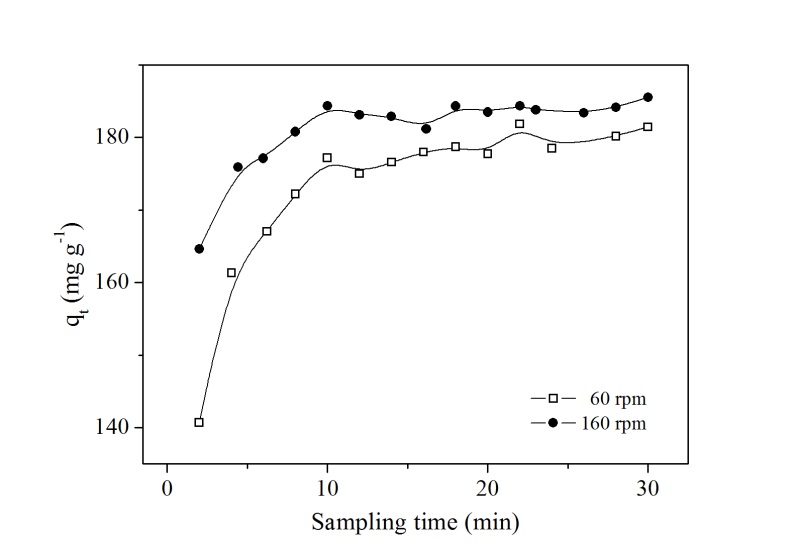


**(e)**

**Fig. 7S** **(a)** Effect of sampling time and initial MB concentrations on adsorption; **(b)** Effect of sorbent dose on q_e_ and dye removal percentages; **(c)** Effect of solution pH on dye removal percentages; **(d)** Effect of solution salt on dye removal percentages and **(e)** Effect of shaking speed on adsorption process.

## Appendix 8. Confocal microscopy (CLSM) to observe periphyton in PPBR

In order to further understand the distribution of MB molecule in periphyton and to understand the mechanism of dye purification process, we used confocal microscopy (CLSM) to observe periphyton in periphyton-based photo-bioreactor (PPBR). Confocal microscopy can be used to colorize various components of the periphyton, while avoiding the destruction of the periphyton structure. Considering MB is a fluorescent substance, periphyton after contact with MB was observed by CLSM technique, and the related images were scanned to reflect the distribution of MB molecules on the surface of periphyton. The following figure shows CLSM image of periphyton in PPBR.


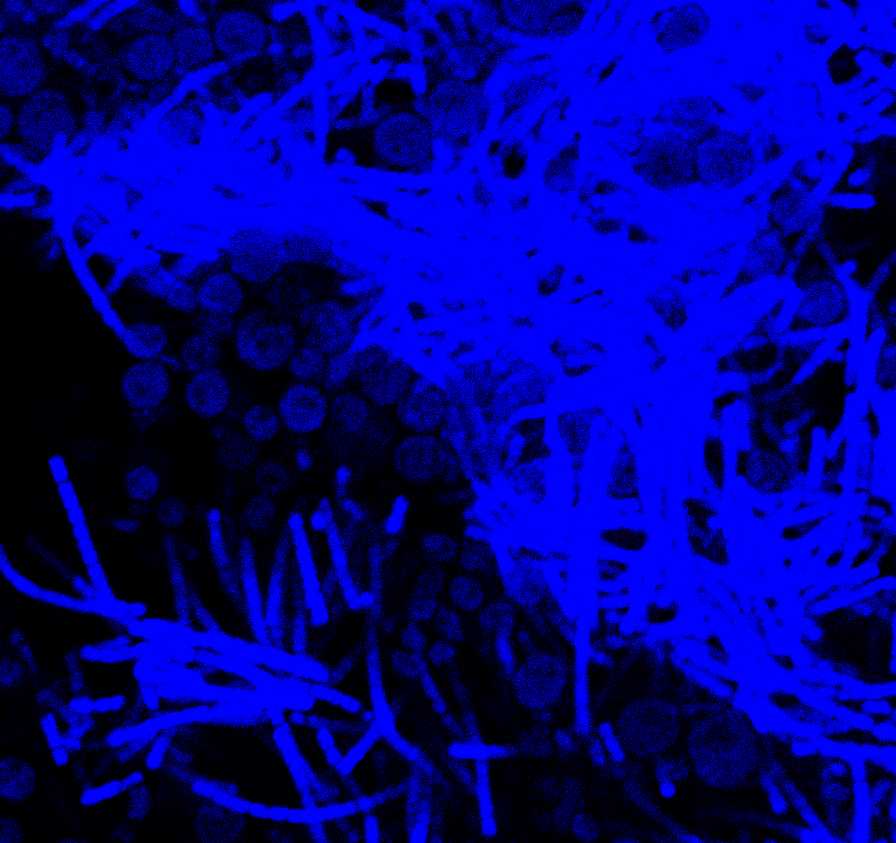


**Figure 8S.** Confocal microscopy photography of MB-treated periphyton in PPBR

(× 630, oil immersion objective, Characteristic fluorescence of MB)

It can be found that MB characteristic fluorescence (blue area) is mainly observed on the surface of the elongated organism, and the elongated organisms are bar-shaped algae as well as attached extracellular polymers (EPS). Combined with microscopic observation and expert identification, the dominant alga is ***Anabaena***. MB aggregates on the surface of *Anabaena* cells and their EPS, indicating that *Anabaena* may play a crucial role in the degradation of MB. At the same time, taking into account the biomass of algae in the cultivated periphyton is much larger than microbes, therefore, it can be speculated that the microalgae (mainly *Anabaena*) played a major role in MB decolorization process. According to previous reports, some microalgae such as Chlorella vulgaris, Lyngbya lagerlerimi,Nostoc lincki, Oscillatoria rubescens, Elkatothrix viridis and Volvox aureus were applied to decolorize and remove basic cationic dyes. And these algae showed different efficiency for dye removal; varied from ∼4 to 95% according to algal species, growth conditions and dye molecular structure [^1^](#_ENREF_1). This indicates that microalgae have decolorization ability on dyes and are closely related to different algae species. The decolorization activity of microalgae is achieved via different mechanisms, partially by certain enzymes implicated in degradation of these compounds.

This study may be the first time observed that *Anaeriana* has degradation effect on highly toxic cationic dye (MB).

## Appendix 9. Nomenclature

| C_0_ initial MB concentration (mg L^-1^)  C_t_ MB concentration at time t (mg L^-1^)  ΔG^0^ Gibbs free energy change (kJ mol^-1^)  ΔH^0^ enthalpy change (kJ mol^-1^)  ΔS^0^ entropy change (J mol^-1^ K^-1^)  k_2_ pseudo-second-order kinetic model rate constant (mg g^-1^ min^-1^)  K_F_ Freundlich adsorption constant (mg g^-1^)  K_id_ intraparticle rate constant (mg g^-1^ min^-0.5^)  K_YN_  the rate constant of Yoon-Nelson model (min^-1^)  m mass of adsorbent (g)  n Freundlich constant  q_e_ the amount of dye adsorbed onto the adsorbents at equilibrium (mg g^-1^)  q_e,cal_ the amount of dye adsorbed onto the adsorbents calculated by model at equilibrium (mg g^-1^)  q_t_ the amount of dye adsorbed onto the adsorbents at time t (mg g^-1^)  q_m_ the maximum adsorption capacity for adsorbent (mg g^-1^)  q_m,cal_ the maximum adsorption capacity calculated by Langmuir model (mg g^-1^)  q_0YN_ the adsorption capacity of the column (mg g^-1^)  Q the flow rate of the column (mL min^-1^)  R ideal gas constant (8.314 J mol^-1^ K^-1^)  *R^2^* linear regression coefficient  t time (min)  τ the time required for 50% dye breakthrough in column study (min)  T temperature (K)  V the volume of solutions (L) |
| --- |

## Reference
